# Development and Evaluation of a Tailored Mobile Health Intervention to Improve Medication Adherence in Black Patients With Uncontrolled Hypertension and Type 2 Diabetes: Pilot Randomized Feasibility Trial

**DOI:** 10.2196/17135

**Published:** 2020-09-23

**Authors:** Antoinette Schoenthaler, Michelle Leon, Mark Butler, Karsten Steinhaeuser, William Wardzinski

**Affiliations:** 1 Department of Population Health NYU school of Medicine, Center for Healthful Behavior Change NYU Langone Health New York, NY United States; 2 Department of Clinical Psychology Fordham University New York, NY United States; 3 Center for Personalized Health Feinstein Institutes for Medical Research Northwell Health Manhasset, NY United States; 4 Radiant Point Technologies, Inc. Honolulu, HI United States

**Keywords:** mHealth, medication adherence, hypertension, type 2 diabetes, African Americans

## Abstract

**Background:**

Research has underscored the need to develop socioculturally tailored interventions to improve adherence behaviors in minority patients with hypertension (HTN) and type 2 diabetes (T2D). Novel mobile health (mHealth) approaches are potential methods for delivering tailored interventions to minority patients with increased cardiovascular risk.

**Objective:**

This study aims to develop and evaluate the acceptability and preliminary efficacy of a tailored mHealth adherence intervention versus attention control (AC) on medication adherence, systolic blood pressure (SBP), diastolic blood pressure (DBP), and hemoglobin A_1c_ (HbA_1c_) at 3 months in 42 Black patients with uncontrolled HTN and/or T2D who were initially nonadherent to their medications.

**Methods:**

This was a two-phase pilot study consisting of a formative phase and a clinical efficacy phase. The formative phase consisted of qualitative interviews with 10 members of the target patient population (7/10, 70% female; mean age 65.8 years, SD 5.6) to tailor the intervention based on the Information-Motivation-Behavioral skills model of adherence. The clinical efficacy phase consisted of a 3-month pilot randomized controlled trial to evaluate the tailored mHealth intervention versus an AC. The tablet-delivered intervention included a tailoring survey, an individualized adherence profile, and a personalized list of interactive adherence-promoting modules, whereas AC included the tailoring survey and health education videos delivered on the tablet. Acceptability was assessed through semistructured exit interviews. Medication adherence was assessed using the 8-item Morisky Medication Adherence Scale, whereas blood pressure and HbA_1c_ were assessed using automated devices.

**Results:**

In phase 1, thematic analysis of the semistructured interviews revealed the following 5 major barriers to adherence: disruptions in daily routine, forgetfulness, concerns about adverse effects, preference for natural remedies, and burdens of medication taking. Patients recommended the inclusion of modules that address improving patient-provider communication, peer vignettes, and stress reduction strategies to facilitate adherence. A total of 42 Black patients (23/42, 55% male; mean age 57.6 years, SD 11.1) participated in the clinical efficacy pilot trial. At 3 months, both groups showed significant improvements in adherence (mean 1.35, SD 1.60; *P*<.001) and SBP (−4.76 mm Hg; *P*=.04) with no between-group differences (*P=*.50 and *P=*.10). The decreases in DBP and HbA_1c_ over time were nonsignificant (−1.97 mm Hg; *P*=.20; and −0.2%; *P*=.45, respectively). Patients reported high acceptability of the intervention for improving their adherence.

**Conclusions:**

This pilot study demonstrated preliminary evidence on the acceptability of a tailored mHealth adherence intervention among a sample of Black patients with uncontrolled HTN and T2D who were initially nonadherent to their medications. Future research should explore whether repeated opportunities to use the mHealth intervention would result in improvements in behavioral and clinical outcomes over time. Modifications to the intervention as a result of the pilot study should guide future efforts.

**Trial Registration:**

ClinicalTrials.gov NCT01643473; http://clinicaltrials.gov/ct2/show/ NCT01643473

## Introduction

### Background

Despite advances in treatments for hypertension (HTN) and type 2 diabetes (T2D), Black patients continue to experience disproportionately lower rates of blood pressure (BP) and glycemic control than those observed in White patients [1,2]. Poor medication adherence among Black patients may explain the disproportionately lower rates of BP and glucose control in this patient population than in White patients [3,4]. Compared with their White counterparts, Black patients with HTN and T2D have been shown to be 1.81 to 4.30 times less likely to adhere to their medication regimen [4-6]. Given that a sufficiently high level of adherence is key for achieving adequate disease control, it follows that successful approaches to reducing the racial gap in cardiovascular-related mortality that exist between Black and White patients must take into consideration the factors driving poor medication adherence in Black population.

Despite a wealth of research dedicated to understanding adherence behaviors in patients with T2D and HTN [3,4], trials designed to improve adherence in minority patients have shown limited effectiveness [7]. Several investigators have called attention to the need for tailored interventions to improve medication adherence in minority patients [7,8], with *tailoring* referring both to cultural tailoring (eg, medication beliefs) and adapting the intervention to match patients’ needs and preferences. Increasingly, mobile health (mHealth) technologies, such as mobile phones, tablets, and other personal digital assistants, are being used as efficient and acceptable methods for delivering tailored interventions to patients with increased cardiovascular risk. Several systematic reviews have documented the short-term benefits of mHealth interventions for improving medication adherence in patients with HTN or T2D [9,10]. However, of the mHealth interventions that have aimed to improve medication adherence, only 6 have been conducted in high-risk minority populations with HTN or T2D, all of which used text messages as their primary method of intervention delivery [11-16]. Although text messaging offers several advantages for improving adherence behaviors (eg, sending reminder prompts in real time), several shortcomings have also been noted. For example, with text messaging, only brief educational, motivational, and/or behavioral content within a limited number of characters can be provided. As a result, they can lack depth by not covering all the necessary content and require individuals to access links for supplementary materials (eg, through videos) [17]. Although personalization is possible with text messaging, qualitative feedback from studies also note that content can become repetitive and predictable, leading to message fatigue and disengagement from the intervention [18,19]. Reading and responding to text messages also require a level of visual acuity and dexterity that may be challenging for people who experience any motor and/or visual impairments. This is especially true for patients with uncontrolled T2D and/or HTN who may experience retinopathy as a result of their disease.

Owing to their portability and ease of use, tablets are increasingly being used as an acceptable digital platform to deliver interventions across all age and racial and ethnic groups [20,21]. Tablet devices offer several advantages over text messages. This includes an adjustable font or icon size, a touchscreen, the ability to integrate video and auditory features into the intervention, which may be better suited for individuals with chronic disease and people with lower levels of lower health literacy, and the ability to create a more interactive and hands-on learning environment.

### Objectives

Consequently, the development of tailored mHealth interventions that use alternative digital platforms to improve medication adherence in Black patients is needed to address the marked racial disparities in BP and glycemic control. In this paper, we report the development and evaluation of an interactive tablet-delivered intervention that was socioculturally tailored for Black patients with uncontrolled HTN and/or T2D who were initially nonadherent to their medications.

## Methods

### Design Overview

We conducted a two-phase feasibility study [22] that included a formative phase and a clinical efficacy phase. The formative phase consisted of qualitative interviews to tailor the intervention to the needs and preferences of the target population. For the clinical efficacy phase, we evaluated the acceptability and preliminary efficacy of the tailored mHealth intervention versus an attention control (AC) condition on changes in medication adherence, systolic BP (SBP), diastolic BP (DBP), and hemoglobin A_1c_ (HbA_1c_) at 3 months (exploratory outcomes) among a sample of 42 Black patients with uncontrolled HTN and/or T2D who were initially nonadherent to their medications in a pilot randomized controlled trial (RCT). We hypothesized that the mHealth intervention would be acceptable and result in better medication adherence and a greater reduction in SBP, DBP, and HbA_1c_ at 3 months compared with the AC group.

### Setting and Participants

This study was conducted at a safety net primary care clinic in New York City, which serves a predominately diverse, low-income urban patient population. Eligibility criteria for both phases of the study included patients who self-identified as Black or African American, received care at the primary care clinic, had uncontrolled HTN defined as BP>140/90 mm Hg (or BP>130/80 mm Hg for those with diabetes or kidney disease) and/or uncontrolled T2D defined as HbA_1c_>7% on at least two visits in the past year and at least one cardiovascular risk factor (eg, hyperlipidemia or obesity), had been prescribed at least one antihypertensive or oral antidiabetic medication and were nonadherent to their medication at screening (as described in the following paragraph), were at least 18 years old, were fluent in English, and did not have significant psychiatric comorbidity. The Institutional Review Board of New York University approved the study.

Potentially eligible patients for both phases were identified through a review of the electronic medical records, after which letters, signed by the physician, were sent to patients inviting them to participate in the study. A trained research assistant (RA) completed all screening, consent, and data collection procedures. During the screening procedures, medication adherence was assessed using the validated 8-item Morisky Medication Adherence Scale (MMAS-8) [23-25]. The first 7 items require a yes or no response, and the final item uses a 5-point scale (*never/rarely* to *all the time*). Total MMAS-8 scores range from 0 to 8, with a score of <6 indicating nonadherence [23]. Only patients with a score <6 were eligible to participate in the study. After obtaining patients’ written informed consent and completion of baseline measures, the statistician randomized eligible patients in a ratio of 1:1 to either the intervention or AC condition using block randomization, with the investigators blinded to the permutation. Following Consolidated Standards of Reporting Trials guidelines [26], the randomization sequence was kept in a secure electronic file that only restricted staff could access. Given the nature of the intervention, patients could not be blinded to the group assignment [27]. However, we used automated BP and HbA_1c_ devices to lower the likelihood that the RA could influence the clinical outcomes.

### Formative Phase: Development of the mHealth Intervention

The Information-Motivation-Behavioral skills (IMB) model of adherence is the theoretical framework underlying the intervention [28,29]. This model views the interrelations between adherence-related information (eg, how medications work), motivation (eg, attitudes or beliefs), and behavioral skills (eg, self-efficacy to take medications) as the fundamental determinants of behavior. A trained qualitative researcher (AS) conducted semistructured interviews in a dedicated, private space with a convenience sample of 10 Black patients before the initiation of the trial. Each interview was approximately 30 min in duration, audiotaped, and professionally transcribed.

The constructs of the IMB model were used to develop the interview questions and probes that were asked in the semistructured interviews. Specifically, questions targeted the most salient informational (eg, how medications work, side effects), motivational (eg, social support, beliefs), and behavioral (eg, self-efficacy, ability to administer medications) barriers and facilitators that may affect adherence behaviors. Interview questions on the most salient barriers to adherence were constructed using the IMB survey items and from the existing adherence literature, including the authors’ research in this patient population [28,30,31]. Sample interview questions included, “What, if any, reasons did your doctor give you about why s/he felt that you needed to take blood pressure/diabetes medicines? (information),” “What, if any, concerns do you have about the medications you are taking for your HTN/T2D? (motivation),” and “Tell me about situations or times that make it more difficult to take your HTN/T2D medications (e.g., when traveling, at work, when costs are too high)? (behavioral skills).” To better fit the needs, beliefs, and experiences of Black patients with HTN and/or T2D, interview questions were also used to identify the sociocultural barriers and facilitators to adherence that were not captured in the survey [32,33]. An example question about sociocultural factors was “Tell me about situations when you have used home remedies to improve your blood pressure/diabetes? What specific home remedies do you take?”

### Description of the mHealth Intervention Group

The mHealth intervention was built by Radiant Point Technologies using Microsoft’s Models, Views, and Controllers Entity Framework as the development environment for the intervention. The intervention consists of an administrative interface for creating user accounts and exporting data and a patient portal for entering information (eg, user profile, questionnaires) and completing activity modules. The fully automated intervention consisted of 3 main parts: (1) a *tailoring survey* based on the IMB Adherence Questionnaire [34], (2) an *individualized adherence profile*, and (3) a personalized list of *interactive adherence-promoting modules* that were matched to the barriers outlined on the adherence profile.

**Figure 1:**
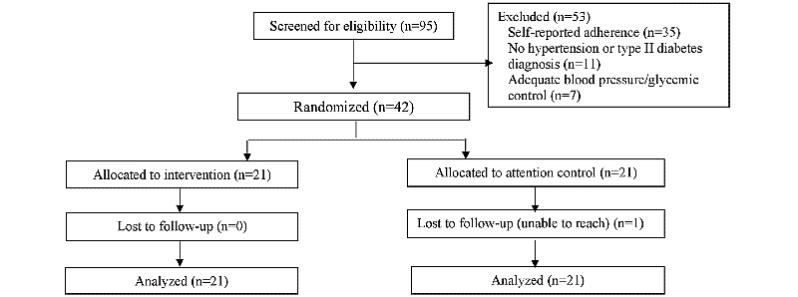
CONSORT (Consolidated Standards of Reporting Trials) flow diagram.

Figure 1 shows the flow of the intervention for participants randomized to the mHealth intervention arm. Once randomized to the intervention group, the RA escorted patients to a private room and provided them with a tablet and instructed them on how to begin the program via a password-protected portal. Patients then completed the IMB tailoring survey, and their responses were immediately scored with an automated algorithm that calculated their 2 most salient adherence barriers (see *Study Measures* section for a description of the scoring algorithm). These data were used to create an individualized adherence profile for each patient in the intervention group, which described the 2 most salient adherence barriers identified by the survey and displayed a personalized list of up to 6 adherence-promoting strategies that were matched to the adherence barriers (Table 1). Patients then had the opportunity to select and work through any (or all) of the strategies that they felt were of the greatest importance and utility for improving their medication adherence. At the end of the program, patients developed an adherence action plan using the principles of specific, measurable, achievable, relevant, and time-bound goal setting. As a feasibility study, patients only interacted with the intervention once at the time of their baseline visit.

**Table 1:** Intervention Information-Motivation-Behavioral constructs and matched adherence-promoting strategies.

Information-Motivation-Behavioral construct and modules			Example intervention strategies
**Information (knowledge about HTN^a^ and medication regimen; side effects and drug interactions)**			
	The City map	Interactive map that allows patient to choose different buildings (eg, hospital, community clinic) that describe local and national services for prescription assistance	
	Pharmacist corner	Interactive prescription label that allows patients to select areas on the label to learn more about what the information means and why it is important	
**Motivation (individual and social) (beliefs or attitudes [ie, illness perceptions, concerns]; social norms or influence; perceived efficacy; depression or stress)**			
	Helping hands	Narratives by Black patients that discuss the importance of taking medications in context of their life values (ie, religious beliefs, family coherence), strategies to talk to their doctor about medications, and how to develop routines to take medications every day Positive voice videos that allow patients to hear about other Black patients’ experiences with HTN and type 2 diabetes and how they overcame challenges to taking medications, as prescribed	
	Relaxation station	Interactive body map that allows patients to learn how common stressors affect their health Guided relaxation activity Discussion on the use of prayer and affirmations to combat the negative effects of stress	
	Doc-Talk	Question building section that allows patients to develop a list of questions they would like to ask at their next visit Tip sheet on how to express concerns about and goals for medications to providers	
**Behavioral skills (habituation and vigilance; routine; ability [subjective and objective])**			
	Myth busters	Interactive game to increase disease- and regimen-specific knowledge as well as address misconceptions or beliefs about medications through a true or false quiz	
	Habit formation	Development of if-then statements that help patients develop habits to take medications even when their routines are disrupted	
	Goal setting	Develop specific, measurable, achievable, relevant, time-bound goals for adherence Celebrate success that allows patients with perfect adherence to create a reward certificate	

^a^HTN: hypertension.

### Description of the AC Group

To control for attention and novelty of the technology, patients randomized to the AC group completed the introductory tailoring survey on the same platform as the patients in the intervention group; however, they did not receive the results displaying their 2 most salient adherence barriers. After the completion of the tailoring survey, the program directed patients to a menu of health education modules on topics unrelated to medication adherence. The duration of the modules was the same as that of the intervention; the modules included basic information derived from the resources published by national organizations (eg, National Cancer Institute) on areas such as the cause and consequences of the disease, associated risk factors, and lifestyle changes.

### Study Measures

#### Acceptability

**Figure 2:**
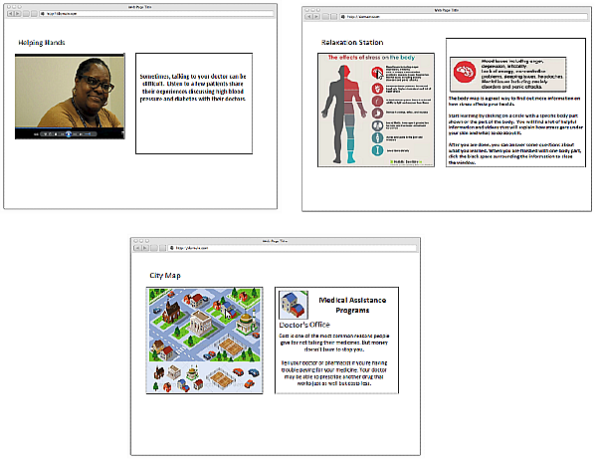
Screenshots of intervention modules.

The acceptability of the intervention was assessed through exit interviews conducted with patients in the intervention group at the 3-month visit. Questions inquired about the perceived ease of use of the tablet-delivered intervention, usefulness of the intervention to address patients’ adherence barriers, relevance of the content, satisfaction with the different intervention modules (Figure 2), and recommendations for improvement.

#### Preliminary Efficacy

The preliminary efficacy of the intervention was assessed at baseline and 3 months using behavioral and clinical outcome measures of medication adherence, reduction in SBP and DBP, and reduction in HbA_1c_. A 3-month follow-up was chosen to mimic clinical practice for measuring changes in BP and HbA_1c_ in patients with uncontrolled HTN and T2D, respectively.

#### Medication Adherence

Medication adherence was assessed using the well-validated MMAS-8 score (α=.83) [23]. The MMAS-8 has a reported sensitivity of 93% and specificity of 53% in detecting nonadherence when compared with prescription refill data [23]. Higher scores on the MMAS-8 have been associated with higher rates of uncontrolled BP and poor glycemic control among adults with HTN and T2D, respectively [23].

#### BP

BP was assessed using validated automated WatchBP monitors (Microlife) at all study visits, following the American Heart Association guidelines [35]. The average of 3 SBP and DBP readings was used as the measurement for each study visit.

#### HbA_1c_

HbA_1c_ was assessed using a blood sample drawn via finger-stick and analyzed using a validated point-of-care device (Afinion AS100 Analyzer) that provides HbA_1c_ results in 3 min.

#### IMB Tailoring Survey

Constructs of the IMB model in the tailoring survey were assessed using a modified version of the empirically validated IMB Adherence Questionnaire [36]. The scale was originally designed to measure the barriers and facilitators of adherence among patients who were HIV positive in clinical care. The 33-item questionnaire comprises 3 subscales, which quantify patients’ adherence-related informational (9 items), motivational (10 items), and/or behavioral skills (14 items) and strengths and weaknesses. Responses were given on a 5-point Likert scale, ranging from *I strongly* disagree to *I strongly agree.*

The barriers identified in the IMB survey were used to create individualized adherence profiles that drove the intervention content. Responses given in a *critical range* for each subscale of the questionnaire (ie, response of strongly agree) reflected a significant deficit in the adherence behavior. Automated decision rules and algorithms were used to synthesize the data and generate an individualized adherence profile that summarized the patient’s barriers to medication adherence from greatest to least (scores range from 0% [no problem] to 100% [significant problem]). The most salient barriers for the adherence profile were those with the top 2 relatively higher scores than those with other barriers.

#### Other Assessments

The RA abstracted clinical data from patients’ electronic health record at the initial screening visit and 3-month visit, including duration of HTN and T2D, total number and classes of antihypertensive and oral antidiabetic medications, and comorbid conditions. Data on patient sociodemographics, including age, gender, household income, education level, employment status, and health insurance status were collected from patients at baseline.

#### Health Literacy

Health literacy was also assessed at baseline using the 36-item short-form Test of Functional Health Literacy in Adults (s-TOFHLA) [37]. The s-TOFHLA is a reading comprehension test that has been linked to poorer health outcomes in racial and ethnic minority populations [37-39]. Total scores of 0 to 16 indicate inadequate health literacy, 17 to 22 indicate marginal health literacy, and 23 to 36 indicate adequate health literacy. Health literacy was included as a covariate in all analytic models.

### Analysis

Sample size estimates for the formative phase were based on best practices for maximizing the information power of qualitative research, which recommends beginning with 8 to 10 participants and adding to the sample, as needed [40]. All interviews were audiotaped and transcribed verbatim. Two members of the study team trained in qualitative methods conducted the analysis of the audiotaped interview data. The transcripts of the interviews were uploaded to the Atlas.ti program to facilitate coding and analysis. The transcripts were individually reviewed and analyzed using the grounded theory constant comparison method [41,42]. Specifically, transcripts were coded line by line using open coding (comparing and categorizing data to generate concepts), axial coding (reorganizing data into categories based on relationships within and between these categories), and selective coding (identifying and describing the central themes to generate a conceptual framework) according to facilitators and barriers to medication taking (eg, side effects, cost, forgetfulness, quality of life). Once the transcripts were independently coded, the research team met to discuss the coding and resolve any discrepancies.

As a pilot trial, our sample size estimates for the RCT were exploratory and intended to generate pilot data to calculate the effect sizes needed for a larger trial. On the basis of meta-analyses of adherence interventions, the sample size was calculated using a moderate change in adherence (0.49 between-group difference) [43] as the effect size, power of .80, and significance level of α of .05. This suggested a sample size of 40 patients (20 per group).

Independent *t* tests and chi-square statistics were used to determine if there were any significant differences between consenting participants who dropped out of the study versus completers on any sociodemographic or clinical variables. To assess acceptability, exit interviews were analyzed using grounded theory methods, as described in the *Formative Phase* section. The analyses of the RCT outcomes were performed using an intent-to-treat design. Analysis of covariance models were used to analyze continuous medication adherence, BP, and HbA_1c_ outcomes measured at baseline and 3 months while controlling for baseline values of each outcome measure in their respective models. The outcomes were modeled as functions of time, treatment, and time-by-treatment interaction. Missing data were handled by estimating model parameters for each individual using maximum likelihood estimation based on the available data.

## Results

### Formative Phase

We invited 13 patients with HTN and/or T2D (4 men and 9 women) to participate in the interviews, of which 3 declined to participate, leaving a total of 10 patients. The reasons for declining participation included being too busy and not being interested in participating in research. Of the 10 patients who agreed to participate, 70% were female, and the mean age was 65.8 years (SD 5.6). The participants varied in their use of technology. Overall, 30% (3/10) of participants exclusively used mobile phones for the primary purposes of talking with family and friends and setting alarms and alerts. Half of the participants used both tablets and mobile devices most commonly to communicate with others and play games. A minority of participants (2/10, 20%) used their devices to track their health (eg, to track medication taking or doctor’s appointments).

On the basis of qualitative feedback from the interview participants, we made several changes to the wording of the IMB survey to reflect a sample of patients with HTN hypertensive and T2D. For example, the question, “I know how my HIV medications interact with alcohol and street drugs” was revised to state, “I know how my [high blood pressure/diabetes] medications interact with other over the counter medications like cough and cold medicines.”

The analysis of the interviews revealed 5 major barriers to adherence: (1) disruptions in daily routines, (2) forgetfulness, (3) concerns about adverse effects, (4) preference for natural remedies, and (5) burdens of medication taking. Specifically, interviewees commented on the challenges of remembering to take their medications when rushing in the morning, traveling, attending appointments, or experiencing other disruptions to their daily routine. Several interviewees also expressed concerns about the side effects of the medications and the potential long-term harm that they may cause to their body. These fears sometimes caused interviewees to “take breaks” from their medications to “let their bodies heal.” They also preferred taking natural remedies to treat their HTN and T2D because “all medications, to some degree, are toxic and not from the Earth.” Finally, despite acknowledging the need for medications, all the interviewees felt that their life is *limited* because of the medications. For example, one interviewee commented that feeling dependent on insulin constrains their ability “to travel, be active, and just have fun.” The short- and long-term concerns about medications expressed by the participants in the interviews (ie, themes 3-5) were noted as key sociocultural beliefs to address in the intervention to improve adherence behaviors in Black patients.

**Figure 3:**
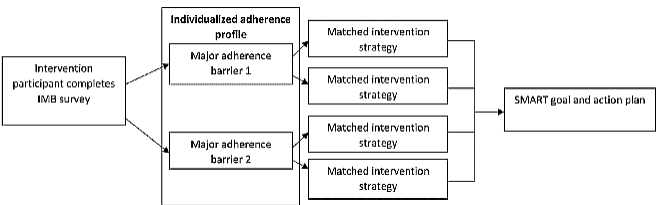
Intervention flow. SMART: specific, measurable, achievable, relevant, time-bound.

In total, 3 strategies to promote adherence also emerged from the interviews: collaborative patient-provider communication, use of peer vignettes, and stress reduction techniques. Overall, most interviewees trusted their providers but felt that they lacked the skills to confidently speak with him or her about their medication concerns. They recommended including interactive modules that allowed them to prepare a list of questions they could ask their provider to help facilitate this discussion. Peer vignettes about common challenges others face in managing their HTN and T2D was also a commonly discussed strategy to improve medication adherence. Interviewees recommended including videos featuring peers who share their experiences overcoming challenges to make healthy lifestyle changes to improve their HTN and T2D and tips for integrating medication taking into their daily routine. Finally, all interviewees commented on the importance of including modules that explain how stress affects health and learning stress reduction techniques to improve their emotional and physical well-being. On the basis of these themes, we developed a list of evidence-based adherence-promoting strategies that could help patients address the aforementioned barriers (Table 1 and Figure 3).

Each strategy was socioculturally tailored for Black patients based on feedback from the interviews, the research teams’ expertise, and the existing literature [7,30,43-47]. For example, the Myth Busters module addressed inaccurate beliefs about medications by including true or false questions about the use of herbal remedies to treat HTN and T2D, the effectiveness of generic medications, and the long-term safety of medications. The Helping Hands video included video testimonials by Black patients who participated in the formative phase interviews and spoke about how they overcame difficulties by asking their provider questions about their medications and how their values supported their decision to make healthy lifestyle changes and take medications, as prescribed. Finally, the Relaxation Station discussed the use of prayer and guided meditation as strategies to lower the negative effects of stress on the body.

### Clinical Efficacy Phase

From July 2016 to January 2018, we screened 95 patients for eligibility. Of these patients, we excluded 53 because they did not have an HTN and/or T2D diagnosis (n=11), their condition was under control (n=7), or they reported being adherent to their medications (n=35). Thus, 42 patients participated in the pilot RCT, of which 21 were randomized to each arm (Figure 1). Overall, 42 participants completed the 3-month visit; one patient from the AC group was lost to follow-up. There were no differences between patients who completed the trial and those who were lost to follow-up in terms of any sociodemographic or clinical characteristics (*P*>.05).

The mean age was 57.6 years (SD 11.1), 54.8% were men, 86% (36/42) had an income of ≤US $40,000 per year, and 55% (23/42) had a high school education (Table 2). Approximately half of the patients (19/42, 45%) had Medicaid. On average, patients were prescribed 3.2 (SD 1.8) antihypertensive and oral diabetic medications. In addition, 95% (40/42) of the patients had adequate health literacy. Patients in the intervention group were significantly more likely to be unemployed than patients in the AC group (20/42, 95% vs 9/42, 43%; *P*<.001); thus, employment status was entered as a covariate in all analyses.

For both patients with HTN and T2D, negative attitudes or beliefs about medications (motivation-attitude) and greater personal concerns about taking medications (motivation-personal) were the most salient barriers to medication adherence identified by the IMB tailoring survey at baseline (see Multimedia Appendix 1 for data on all the barriers). On the basis of these barriers, the most frequently matched intervention strategies were the *Helping Hands* vignettes (9/21, 43%), the *Myth Busters* game (5/21, 24%), and the *Doc-Talk* activity (3/21, 14%; Table 1).

**Table 2:** Baseline sociodemographic characteristics for all patients and by study group.

Characteristics		All patients (N=42)		Intervention patients (n=21)		Attention control patients (n=21)	
	Age (years); range: 36-82, mean (SD)		57.6 (11.1)		59.7 (10.7)		54.5 (11.3)
	Gender (male), n (%)		23 (54.8)		11 (52.4)		12 (57.1)
**Marital status, n (%)**							
	Single		11 (26.2)		5 (23.8)		6 (28.6)
	Married		8 (19.0)		3 (14.3)		5 (23.8)
	Divorced or separated		16 (38.1)		9 (42.9)		7 (33.3)
	Widowed		7 (16.7)		4 (19.0)		3 (14.3)
**Education, n (%)**							
	Less than high school		5 (11.9)		2 (9.5)		3 (14.3)
	High school or technical school		23 (54.8)		13 (61.9)		10 (47.6)
	Some college		6 (14.3)		3 (14.3)		3 (14.3)
	College and above		8 (18.8)		3 (14.3)		5 (23.8)
	Unemployed		29 (69.0)		20 (95.2)		9 (42.9)
**Income (US$), n (%)**							
	<20,000		26 (62.8)		13 (61.9)		13 (61.9)
	20,000-40,000		10 (23.2)		6 (28.5)		4 (19.0)
	>40,000		6 (14.0)		2 (9.5)		4 (19.0)
**Insurance, n (%)**							
	Private		8 (19.0)		3 (14.3)		5 (23.8)
	Medicare without Medicaid		7 (16.7)		4 (19.0)		3 (14.3)
	Medicaid only or with Medicare		19 (45.3)		10 (47.7)		9 (42.9)
	None		8 (19.0)		4 (19.0)		4 (19.0)
**Health literacy, n (%)**							
	Inadequate		1 (2.3)		0 (0)		2 (9.5)
	Marginal		1 (2.3)		0 (0)		1 (4.8)
	Adequate		40 (95.4)		21 (100)		18 (85.7)
Diabetes, n (%)		31 (72.1)		17 (77.3)		14 (66.7)	
Stroke, n (%)		6 (14)		3 (14.3)		3 (14.3)	
Kidney disease, n (%)		2 (4.7)		2 (9.5)		0 (0)	
Number of antihypertensive or oral diabetic medications, mean (SD)		3.20 (1.8)		3.65 (1.8)		2.75 (1.7)	

#### Acceptability of Intervention

Overall, 92% (19/21) of the patients in the intervention group agreed that the mHealth intervention could be an effective tool to help patients take their medications. Specifically, they benefited by learning the importance of taking their medications (17/21, 82%), learning how to speak to their doctor about medication concerns (14/21, 67%), and developing new habits to take medications regularly or make healthy lifestyle changes (19/21, 92%). Most (19/21, 92%) patients rated the vignettes as the best intervention strategy. All patients (21/21, 100%) felt that the intervention was designed for someone like them. The patients suggested the following modifications: (1) shortening the tailoring survey so more time can be spent using the strategies (9/21, 43%), (2) share the results with their doctor to help stimulate conversations about challenges to adherence during the clinic visit (17/21, 82%), and (3) improve tablet button size and sensitivity and audio quality (8/21, 40%). Finally, one-third of patients (7/21, 33%) recommended adding a health educator in addition to the mHealth intervention because they felt it would be beneficial to also discuss questions with a person.

#### Effect of the Intervention on Medication Adherence

The mean self-reported adherence for the intervention and AC groups at baseline was 4.4 (SD 1.3) and 4.0 (SD 1.3, range: 0-8), respectively. There was a significant improvement in adherence across the 3-month study for both groups (mean change 1.4, SD 1.6; *P*<.001). At 3 months, 63.2% of the intervention group compared with 55.6% of the AC group reported being adherent to their medications (MMAS-8 score≥6); however, there were no between-group differences (*F*_1,36_=0.5; *P*=.50; Table 3).

**Table 3:** Change in medication adherence blood pressure and hemoglobin A1c between baseline and 3 months by study group.

Outcome	Control participants (n=21)			Intervention participants (n=21)			*F* test^a^ (*df*)		*P* value
	Baseline	3 months	Baseline		3 months				
Medication adherence, mean (SD); range: 0-8^b,c^	4.0 (1.3)	5.5 (2.1)	4.4 (1.3)		5.6 (2.0)	0.5 (1, 4)		.50	
Change	N/A^d^	1.5	N/A		1.2	N/A		N/A	
Systolic BP^e^, mean (SD) mm Hg	137.4 (17.8)	135.1 (19.5)	139.9 (18.3)		130.9 (17.4)	3.1 (1, 26)		.10	
Change	N/A	−2.3	N/A		−9.0	N/A		N/A	
Diastolic BP, mean (SD) mm Hg	88.5 (10.9)	87.4 (10.3)	84.1 (14.1)		80.2 (16.0)	2.9 (1, 27)		.10	
Change	−1.1	N/A	−3.8		N/A	N/A		N/A	
Hemoglobin A_1c_, n (%)	7.3 (2.8)	7.8 (2.5)	8.5 (3.0)		8.2 (2.7)	1.1 (1, 30)		.30	
Change	N/A	+0.5	N/A		−0.3	N/A		N/A	

^a^*F* statistic results of the analysis of covariance.

^b^Higher scores indicate better adherence.

^c^The eight-item Morisky Medication Adherence Scale (MMAS-8) scoring and coding presented in the study was done using the electronic Morisky Widget MMAS-8 software. The use of the Morisky Widget MMAS-8 software, copyright registration number TX 8-816-517, is protected by US copyright laws. Permission for use of the Morisky Widget MMAS-8 software is required and was obtained for this research. A license agreement is available from MMAS Research LLC 14725 NE 20th St Bellevue, WA 98007, United States; strubow@morisky.org.

^d^N/A: not applicable.

^e^BP: blood pressure.

#### Effect of the Intervention on BP and HbA_1c_

The mean baseline BP for the intervention group was 139.9 (SD 18.3)/84.1 (SD 14.4) mm Hg and that for the AC group was 137.4 (SD 17.8)/87.4 (SD 10.3) mm Hg. SBP significantly improved over time for the total sample (mean −4.8, SD 16.1 mm Hg; *P*=.04). The intervention group showed a 6.7 mm Hg greater reduction in SBP than the AC group, with no between-group difference (*F*_1,26_=3.1; *P*=.10). The reduction in DBP across the 3 months for the total sample was nonsignificant (mean −1.97, SD 9.19 mm Hg; *P*=.20); however, the intervention group showed a 2.7 mm Hg greater reduction in DBP than the AC group.

The mean HbA_1c_ was 8.2% (SD 2.7) in the intervention group and 7.8% (SD 2.5) in the AC group. The decrease in HbA_1c_ across the 3 months for the total sample was nonsignificant (mean −0.2%, SD 0.3; *P*=.50). However, the intervention group exhibited a 0.3% reduction in HbA_1c_ over time, whereas the AC group showed a 0.5% increase.

## Discussion

### Principal Findings

This feasibility study evaluated the acceptability and preliminary efficacy of a theory-driven mHealth intervention that was socioculturally tailored for Black patients with uncontrolled HTN and/or T2D to address their most salient barriers to medication adherence. Exit interviews demonstrated high acceptability of the intervention with patients rating it easy to use, enjoyable, and beneficial for understanding the importance of being adherent and for learning strategies to talk to their providers about medication concerns and developing habits to routinely take their medications and make lifestyle changes. Despite high acceptability, one-third of intervention participants recommended including a health educator as an adjunct to the mHealth intervention, suggesting that some in-person contact is important and could not be replaced by the design of this intervention. Future research should test whether inclusion of an avatar-narrator who uses a storytelling approach to guide patients through the program and answer questions may be a suitable alternative to the educator.

Contrary to our hypotheses, we did not observe significant between-group differences in self-reported medication adherence and SBP reduction at 3 months. However, the results did show that patients in the intervention group exhibited greater improvements in BP and HbA_1c_ across the 3-month study than patients in the AC group. Future research should replicate this study with a larger sample size for a longer duration to determine whether these effects are of clinical significance and can be sustained over time.

Our findings are similar to those of previous studies, which found that tablet-based interventions may be an acceptable approach for addressing medication nonadherence in patients with cardiovascular diseases and T2D [48-50]. However, many of the interventions were medication management systems that provided medication reminders, similar to text messaging programs, and did not address motivational or behavioral barriers to adherence [51,52]. One exception was My Interventional Drug-Eluting Stent Education App (MyIDEA), which was a tablet-delivered intervention designed to improve antithrombotic medication adherence among 24 patients who had a percutaneous coronary intervention [49]. The MyIDEA program combined tailored information about patients’ symptoms with patient vignettes about the importance of medication adherence. Usage data, measured as the time using the MyIDEA program, suggested that the use of patient vignettes was an acceptable intervention approach for this study population. Similar to this study, the intervention group also demonstrated a greater, albeit nonsignificant, increase in medication adherence than the control group.

There are several strengths to this study. First, we included the target population in the design of the intervention to ensure that the end product incorporated the needs, skills, and preferences of the users. Second, the intervention moved beyond relying on a *single bullet approach* to improve medication adherence by using individualized profiles that identified patients’ most salient adherence barriers and subsequently matched the appropriate mix of strategies to address those needs. Finally, we limited our population to nonadherent patients, thereby targeting high-risk patients who are more likely to be high users of the health care system because of uncontrolled T2D or HTN and its related complications.

### Limitations

Despite these strengths, there are several reasons for the null findings of our study. These may include the small sample size and short time frame. Moreover, the use of an AC condition may have served as an intervention itself, thereby diminishing our ability to find between-group differences. We may have also failed to intervene on other important barriers to adherence that were not captured by the IMB survey or identified through feedback from participants during the formative phase of the study. Although this study comprised patients with HTN or T2D, small sample sizes in each disease state (6 patients only had a diagnosis of HTN and 5 only had a diagnosis of T2D) prohibited testing the effectiveness of the intervention in either of these subgroups. However, in the exit interviews, several participants spoke about the relative importance of the 2 diseases, often regarding T2D as more dangerous and thus considered it more important to get under control than HTN. A similar finding was documented in a qualitative study of racially diverse patients with comorbid T2D and HTN [53]. Future research should explore whether additional intervention strategies are needed to address patients’ perceptions about the importance of BP control when also diagnosed as having T2D.

The intervention was also delivered only once; thus, we do not know if a higher dose would have been acceptable or led to improved outcomes. Future research should examine whether implementing the intervention in the clinic waiting room as part of regular care (eg, every 3 months for patients with uncontrolled disease) would help to prepare patients to discuss challenges with medication adherence with their provider and lead to improvements in patient activation, medication adherence, and disease control [54,55]. Finally, it is possible that participants exhibited a recall bias when rating the acceptability of the intervention because these questions were asked 3 months after the completion of the intervention. The methodological limitations of our study are similar to those documented above and in systematic reviews of mHealth interventions targeting medication adherence and reinforce the call for more methodologically rigorous studies of mHealth interventions that include larger sample sizes, are of a longer duration, and use more robust measures of medication adherence to determine the sustainable impact of these approaches on health behavior change [56]. Medication adherence was also assessed by self-report, which may have resulted in an overestimation of adherence levels. Future studies should use a more objective measure of adherence to confirm our findings. Finally, although medication adherence is a primary contributor to BP and glycemic control, other factors such as changes in lifestyle behaviors may explain our reductions in BP and HbA_1c_ but were not measured in this study.

In conclusion, this feasibility study demonstrates the acceptability of a tailored mHealth adherence intervention for Black patients with uncontrolled HTN and/or T2D. Modifications to the intervention that enhance the technical functions and streamline the IMB questionnaire should guide future evaluation of the intervention in a larger sample.

## Appendix

Multimedia Appendix 1 Frequency of top two adherence barriers identified by the information-motivation-behavioral skills model of adherence survey among intervention participants.

## Abbreviations

AC: attention control
BP: blood pressure
DBP: diastolic blood pressure
HbA_1c_: hemoglobin A_1c_
HTN: hypertension
IMB: Information-Motivation-Behavioral skills model of adherence
mHealth: mobile health
MMAS: Morisky Medication Adherence Scale
MyIDEA: My Interventional Drug-Eluting Stent Education App
RA: research assistant
RCT: randomized controlled trial
s-TOFHLA: 36-item short-form Test of Functional Health Literacy in Adults
SBP: systolic blood pressure
T2D: type 2 diabetes
