# Supplementary material for: Development and Evaluation of a Tailored Mobile Health Intervention to Improve Medication Adherence in Black Patients With Uncontrolled Hypertension and Type 2 Diabetes: Pilot Randomized Feasibility Trial
Source: JMIR Mhealth Uhealth. 2020 Sep 23;8(9):e17135. doi: 10.2196/17135 (PMC7542413; doi:10.2196/17135)
Supplement: Multimedia Appendix 1 [file mhealth_v8i9e17135_app1.docx]

**Multimedia Appendix Table**. Frequency of top two adherence barriers identified by the IMB survey among intervention participants.

| **Adherence Barriers identified by IMB survey (N=21)** | **Primary Adherence Barrier, n (%)** | **Secondary Adherence Barrier, n (%)** |
| --- | --- | --- |
| Information | 1 (1.1) | 1 (2.2) |
| Attitudes (Motivation) | 9 (47.8) | 4 (21.7) |
| Personal Motivation | 3 (15.2) | 11 (55.4) |
| Social Motivation | 2 (6.5) | 1 (3.3) |
| Cost (Behavioral) | 5 (27.2) | 3 (13.0) |
| Behavioral Skills | 1 (2.2) | 1 (4.3) |
